# Trends in alcohol-associated liver disease mortality rates in American Indians and Alaskan Natives

**DOI:** 10.1186/s12889-025-22895-x

**Published:** 2025-06-02

**Authors:** Yousaf Zafar, Isaac M. E. Dodd, Arsalan Zafar Iqbal, Laila Manzoor, Naveed Zafar Iqbal, Kristen D. Dodd, Jan Petrasek

**Affiliations:** 1 University of Mississippi Medical Center, Jackson, MS USA; 2 University of Michigan, Ann Arbor, MI USA; 3 Mississippi College, Clinton, MS USA; 4 Texas Liver Institute, San Antonio, Texas, USA; 5 Department of Medicine, Division of Hospital Medicine, University of Mississippi Medical Center, 2500 N State St, Jackson, MS 39216 USA

**Keywords:** Alcohol-associated liver disease, Chronic liver disease, American Indians and Alaskan Natives, Age adjusted mortality rate, Alcoholism, Health disparities

## Abstract

**Introduction:**

A leading cause of death among non-Hispanic American Indians or Alaskan Natives (AI/ANs), apart from cardiovascular disease and unintentional injuries, is chronic liver disease (CLD). This study analyzed recent trends in AI/AN ALD mortality, given their increased incidence of alcoholic liver disease (ALD) and high burden of CLD.

**Methods:**

This cross-sectional study used data from the Centers for Disease Control and Prevention Wide-Ranging Online Data for Epidemiologic Research (CDC WONDER). Geographic, age and sex-based temporal mortality trends of ALD deaths were analyzed among the AI/AN population in the US from 1999 to 2020. Joinpoint regression analyses determined trends in ALD crude and age-adjusted mortality rates, identifying the annual percent change (APC) in each subgroup.

**Results:**

In 1999–2020, the overall age-adjusted mortality rate (AAMR) among AI/ANs increased significantly from 27.2/100,000 to 88.4/100,000. Although men had a higher mortality rate overall, women had a higher increase in APC (2003–2008 APC was 17.7 [95% Cl: 9.9–26.0] and 2018–2020 APC was 25.3 [95% Cl: 11.4–40.9]) compared to men (1999–2020 APC was 5.8 [95% Cl 4.8–6.8]).

All age groups studied witnessed an increase in AAMR. However, the age group 45–64 had the highest mortality overall in the preceding 2 decades. Non-metropolitan geographic regions had the highest mortality rate (2018–2020 APC of 25.5 [95% Cl: 5.0–50.0]) compared to medium/small or large metro areas. Western and Midwestern US Census regions had the highest mortality rates.

**Conclusions:**

Male sex, age 44–65, and rural dwelling was associated with a greater ALD AAMR in AI/AN populations. Social changes due to the Covid-19 pandemic may have led to increased ALD mortality. Discerning the underlying causality of these associations and examining the impact of the social determinants of health, may represent important opportunities to enhance care for AI/ANs as a vulnerable minority population.

**Supplementary Information:**

The online version contains supplementary material available at 10.1186/s12889-025-22895-x.

## Key questions

*What is already known on this topic* – American Indians or Alaskan Natives (AI/ANs) are the racial/ethnic group most vulnerable to alcohol-associated liver disease (ALD) mortality.

*What this study adds* – This study found that during the Covid-19 pandemic in 2020 there was a significantly increase in mortality rate with the annual percent change greatest in females, younger age groups, and rural areas.

*How this study might affect research, practice or policy* – Targeted, culturally relevant interventions for fast reductions in alcohol-attributed mortalities are needed with further studies to determine whether ALD mortality rates have improved since the end of the pandemic.

## Introduction

Digestive diseases represent more than one-third of the global burden of all prevalent chronic disease, a large majority of which is chronic liver disease [1]. Recently global chronic liver disease (CLD) prevalence has increased which appears to be driven partly by an increase in alcohol-associated liver disease (ALD). Rates of ALD in the US increased by 43% from 2009 to 2015 [2]. Almost half of the mortalities due to CLD in 2016 were attributed to alcohol consumption in a study of the global burden of disease [3, 4]. ALD mortality increased significantly by 22.4% during the Covid-19 pandemic [5]. Among all racial groups in the United States, the largest decline in life expectancy is consistently seen among non-Hispanic American Indians or Alaskan Natives (AI/ANs) [2, 6] who are the US racial/ethnic group most vulnerable to ALD. Few publications have examined recent trends in ALD mortality among AI/ANs around the time of the Covid-19 pandemic.

Identifying trends in the mortality rate of AI/ANs can help highlight areas for targeted interventions. Therefore, this study evaluated demographic and regional differences in the age-adjusted mortality rate (AAMR) related to ALD among AI/ANs in the US.

## Methods

Deidentified data was retrieved from the Centers for Disease Control and Prevention Wide-Ranging Online Data for Epidemiologic Research (CDC WONDER) Multiple Causes of Death database [7] for years 1999 to 2020 using ALD death data derived from International Classification of Diseases-10 codes K70.0-K70.9 [8].

Statistical analyses with regression models were performed using the Joinpoint Trend Analysis Software (v5.0.2) published by the National Institutes of Health National Cancer Institute. Census-standardized AAMRs per 100,000 were standardized to the US population for the year 2000. This was used to assess temporal trends in the average annual percent change (AAPC) of mortality rates, fitting log-linear regression models for trends in AAMRs, and applying the simplest model with a maximum of three join points assuming a Poisson distribution. The final models estimated the annual percent change (APC), AAPC, 95% CIs, and p-values, with statistical significance established at *p* ≤ 0.05. An APC was considered increasing or decreasing if the slope of the mortality change was significantly different from zero in two-tailed *t*-testing. Non-parallel pairwise comparisons were used to assess differences in trends between sex and age groups (ages 25–44, 45–65, and 65 and older). AAPC differences were reported for each group. Mortality trends were also analyzed by urbanization level using the National Center for Health Statistics Urban–Rural Classification Scheme and the 2013 US census classification. Three population categories were created: large metropolitan (≥ 1 million), medium/small metropolitan (50,000–999,999), and non-metropolitan, the latter of which included rural or micropolitan (< 50,000) areas [9]. For comparison within age groups, crude mortality rates (CMRs) were used to report the aforementioned outcomes (Supplemental Table S2).

Strengthening the Reporting of Observational Studies in Epidemiology (STROBE) Statement reporting guidelines were followed. In compliance with the Common Rule [10], informed consent or approval from an institutional review board was not required as the data analyzed was deidentified and publicly available.

### Secondary analyses

This study focused solely on AI/AN trends, but a descriptive analysis comparing the AAMR and APC of ALD to all-cause and cardiovascular disease (CVD) mortality. A separate sensitivity analysis was done to compare observed mortality trends by sex excluding 2020. An additional analysis comparing AI/AN mortality with other racial/ethnic groups to contextualize whether AI/AN populations are uniquely affected was not done since this was published elsewhere on CDC WONDER data [5, 11].

## Results

Between 1999 and 2020, a total of 14,828 deaths were related to ALD in the AI/AN population in the US. AAMR increased from 27.2/100,000 (95% Cl: 24.1–30.3) in 1999 to 88.4/100,000 (95% Cl: 83.9–93.0) in 2020.

### Annual AAMR for ALD by level of urbanization among AI/ANs

**Fig. 1:**
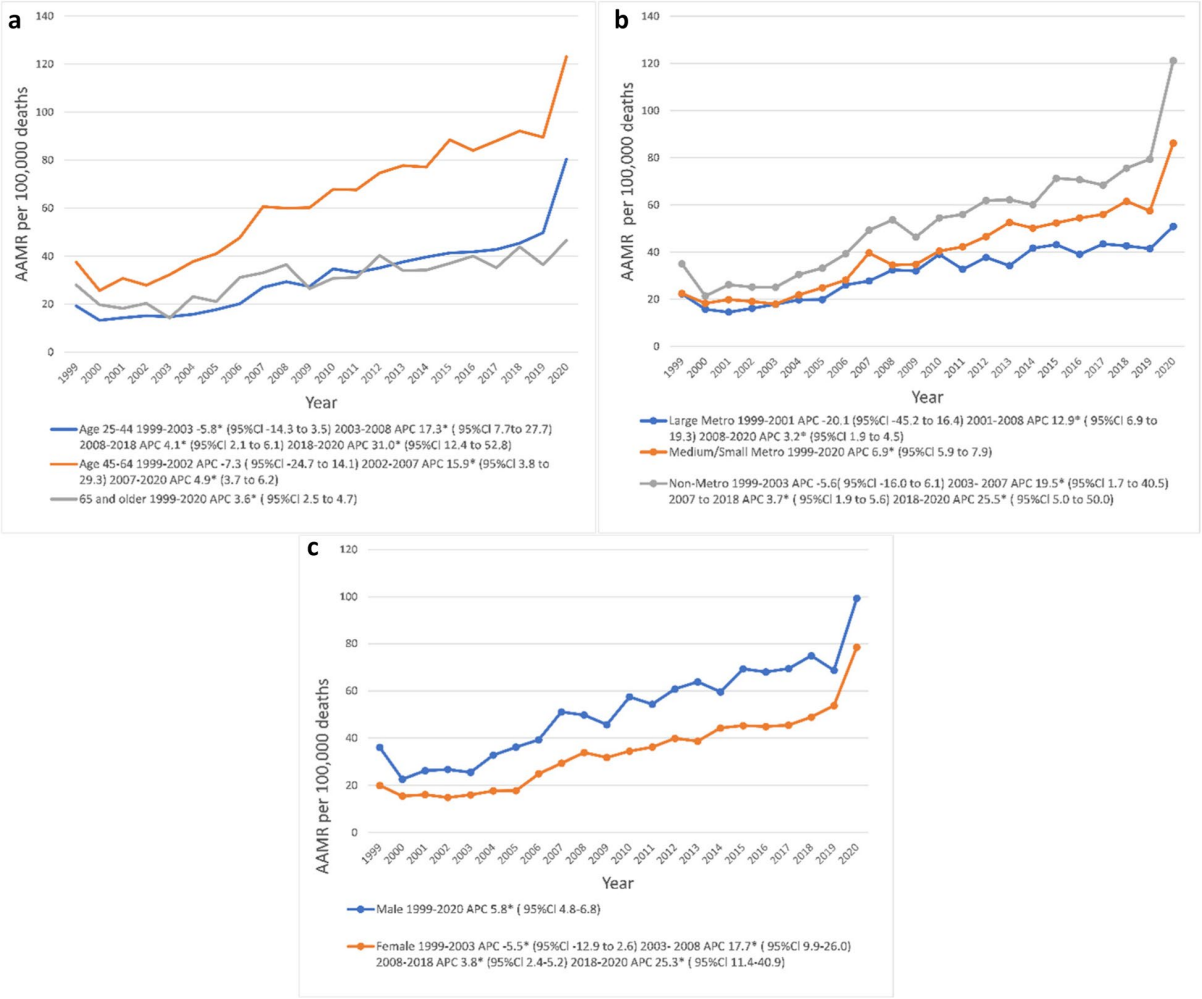
Detailed are line graphs depicting AAMR per 100,000 deaths in the US among AI/ANs by year from 1999–2020 stratified by **a** age groups 25–44, 45–64, and 65 and older years (top left); **b** large, medium/small, and non-metropolitan/rural metropolitan US levels of urbanization (top right); and **c** male and female sex (bottom left). *An asterisk indicates that for the APC marked there is a statistically significant difference from zero at α = 0.05. AAMR = Age-adjusted mortality rate; AI/AN = American Indian or Alaskan Native; APC = Annual percentage change; CI = Confidence interval

Figure 1 shows a steep increase in AAMR for all urbanization levels from 2019 to 2020. In the earlier decades, mortality was always highest in non-metropolitan areas, but after 2019 these areas saw the most pronounced increase in mortality rate by a greater than 33% increase in mortality.

Non-metropolitan areas had consistently higher ALD AAMRs than medium/small or large metropolitan areas throughout the study period, with overall AAMRs being 51.0 (95% CI: 49.3–52.8), 41.7 (95% CI: 40.5–43.0) and 32.4 (95% CI: 31.2–33.5) respectively. For non-metropolitan areas in 1999–2003 the APC initially declined to −5.6 (95% Cl: −16.0 to 6.1) but later increased from 2003 to 2007 with an APC of 19.5 (95% Cl: 1.7 to 40.5). These non-metropolitan areas had a steep increase in APC to 25.5 (95% Cl: 5.0 to 50.0) in 2018–2020. For medium/small metropolitan areas between 1999–2020, the APC was 6.9 (95% Cl: 5.9 to 7.9). For large metropolitan areas the APC later increased in 2001–2008 to an APC of 12.9 (95% Cl: 6.9 to 19.3), but in 2018–2020 the APC had less of an increase at 3.2 (95% Cl: 1.9 to 4.5).

### ALD AAMR stratified by sex among AI/ANs

Overall, there was a higher mortality in males than females from ALD, but in 2003–2008 the increase in APC mortality rate was much higher in females than in males (Fig. 1). For females, AAMR increased from 20/100,000 in 1999 to 78.5/100,000 in 2020. Supplementary Table S1 shows AAMRs stratified by sex. Although there was an initial decline from 1999–2003 for an APC of −5.5 (95% Cl: −12.9 to 2.6), there was afterwards a greater incline in mortality, particularly during 2003–2008 with an APC of 17.7 (95% Cl: 9.9–26.0). There was also a recent significant increase in 2018 to 2020 with an APC of 25.5 (95% Cl: 11.4–40.9). For males, AAMR increased from 36.1/100,000 in 1999 to 99.3/100,000 in 2020, with an AAPC of only 5.8 (95% CI: 4.8–6.8).

### Annual AAMR trend for ALD Stratified by age in AI/ANs

The age group from 45 to 64 had an overall higher mortality with an AAMR of 37.2/100,000 in 1999 and 121.8/100,000 in 2020. For age group 25–44, an initial decline in mortality of −5.8% was seen in 1999 to 2003 followed by a rise in mortality rate in 2003 to 2008 to an APC of 17.3 (95% Cl: 7.7 to 27.7). This was followed by an almost doubled mortality percent change in 2018–2020 to an APC of 31.0 (95% Cl: 12.4 to 52.8). For the 45–64 age group, their AAPCs were steep throughout the study period from 1999–2002 with an APC of −7.3 (95% Cl: −24.7 to 14.1) from 2002 to 2007 where their APC was 15.9 (95% Cl: 3.8 to 29.3). For the age group 65 and older, between 1999 and 2020 the APC remained constant at 3.6 (95% Cl: 2.5 to 4.7) as shown in Fig. 1. Supplementary Table S2 shows crude mortality rates (CMRs) and AAMRs for age subgroups 65–74, 75–84, and aged 85 and over.

### Subgroup analyses of AAMR by geographic region

**Fig. 2:**
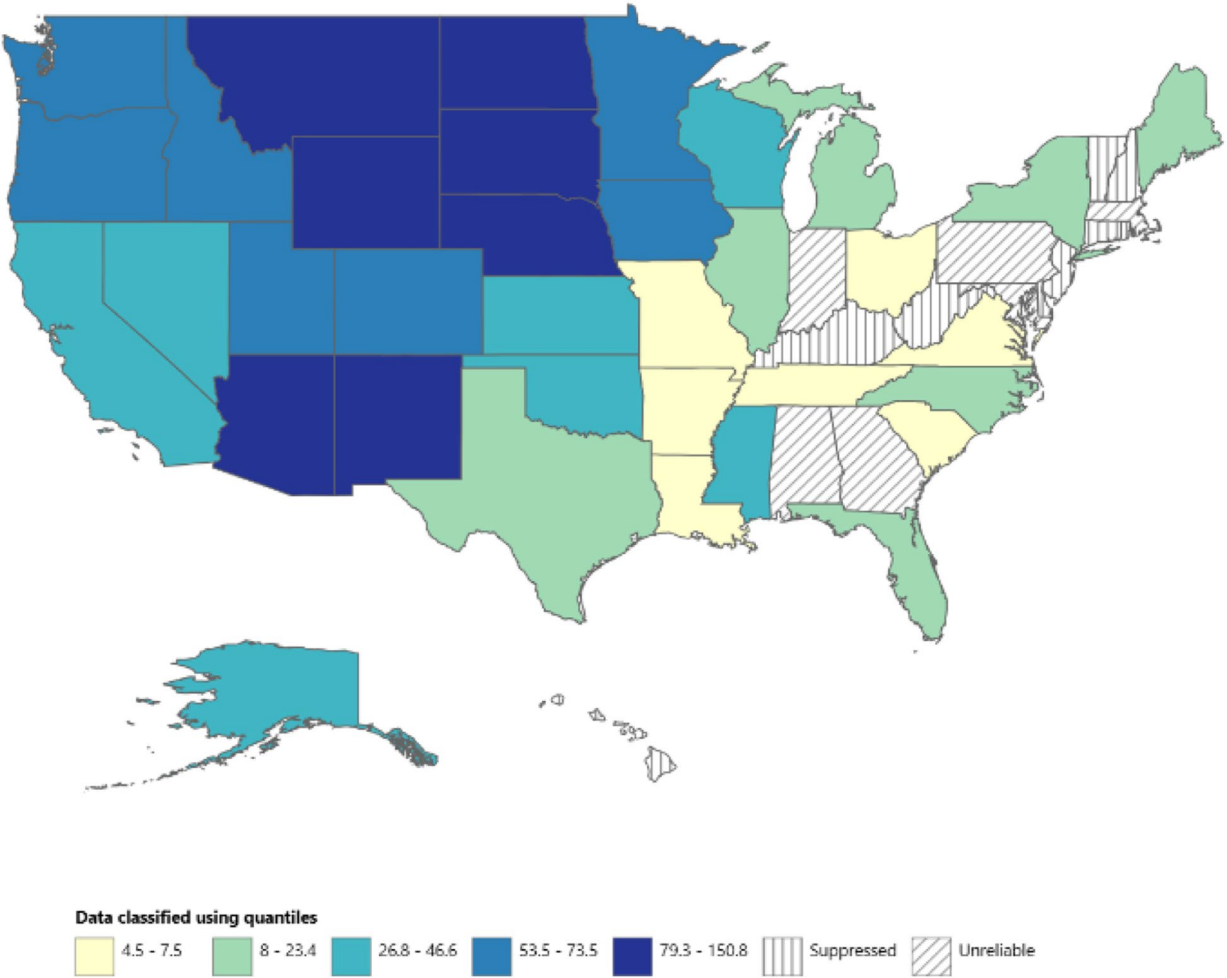
Map of the United States by quantiles of AAMR for ALD among AI/ANs, stratified by state, 1999 to 2020. AAMR = Age-adjusted mortality rate; AI/AN = American Indian or Alaskan Native; ALD = alcohol-associated liver disease; APC = Annual percentage change; CI = Confidence interval

Significant differences in AAMR were observed across states, with their respective AAMRs ranging from 105.4 (95% CI: 91.7–119.1) in Wyoming to 4.5 (95% CI: 2.8–7.0) in Louisiana. States that fell into the top 90 th percentile were New Mexico, Arizona, Wyoming, Montana, North Dakota, South Dakota, and Nebraska (Fig. 2). These states had approximately 10 to 33 times the AAMRs compared with states that fell into the lower 10 th percentile. The lower percentile states were Louisiana, Arkansas, Missouri, Tennessee, South Carolina, Virginia, and Ohio. States that do not have reliable data listed on CDC WONDER are typically when a state’s death count is < 20 for the population being studied. These states included Alabama, Georgia, Indiana, Maryland, Massachusetts, and Pennsylvania. On average, over the course of the study period the highest mortality rate was observed in the West (AAMR of 66.5; 95% CI: 65.1–67.8) followed by the Midwest (AAMR: 49.8; 95% CI: 48.0–51.7), the South (AAMR: 17.7; 95% CI: 16.9–67.8), and the Northeast (AAMR: 10.1; 95% CI: 8.7–11.4) (Supplemental Figure S1).

### Secondary and sensitivity analyses

**Fig. 3:**
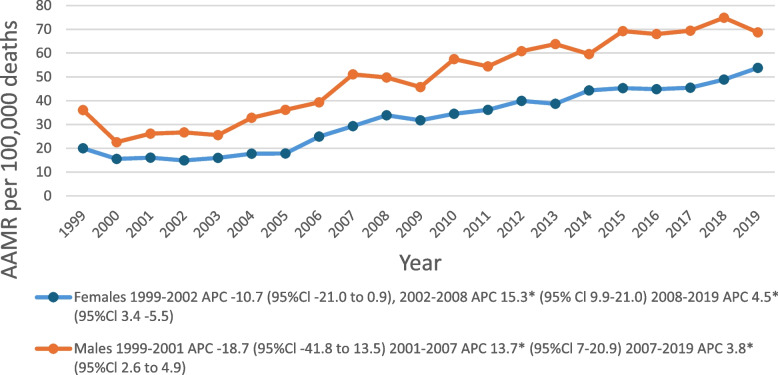
This figure illustrates the results of a sensitivity analysis on AI/ANs by sex as in Fig. 1 but excluding the year 2020. This illustrates that the increase in APC seen in 2018–2020 in Fig. 1 is due primarily to increased ALD mortality in 2020. *An asterisk indicates that for the APC marked there is a statistically significant difference from zero at α = 0.05. AAMR = Age-adjusted mortality rate; AI/AN = American Indian or Alaskan Native; ALD = alcohol-associated liver disease; APC = Annual percentage change; CI = Confidence interval

Due to the findings of increased AAMR APC in 2018–2020, a sensitivity analysis was performed consisting of a repeat joinpoint regression comparing the APC in this interval to the same data but without data from the year 2020 (Fig. 3). AI/AN AAMR per 100,000 deaths but stratified by sex as the chosen independent variable since it is a binary variable very uncommonly misclassified. The joinpoints and intervals were recalculated as an APC of 4.5 (95% CI: 3.4–5.5) in 2008–2019 for females and 3.8 (95% CI: 2.6–4.9) in 2007–2019 for males, the APC in females in the analysis being lower than the APC of 25.3 in females for the same interval of 2018–2020 in the main analyses that included the year 2020.

ALD mortality was compared in a secondary analysis to all-cause and CVD mortality (Supplemental Figure S2). As expected, the CVD mortality rate was higher than ALD mortality, but the APC in 2018–2020 was higher at 16.4 (95% CI: 3.7–30.7) versus 10.7 (95% CI 3.3–18.7).

## Discussion

As demonstrated, the overall prevalence of ALD mortality among AI/ANs has steeply risen. Our cross-sectional study found that AI/AN men had a higher AAMR (88.4 per 100,000) in 2020 than in prior years, but women experienced a sharp increase in AAMR APC at 25.3 (95% CI: 11.4–40.9) in 2018–2020. Overall, the percent change in mortality due to ALD (16.4 [95% CI: 3.7–30.7]) was greater than the percent change in CVD mortality (10.7 [95% CI: 3.3–18.7]) during 2018–2020 which correlates with the Covid-19 pandemic period (Supplemental Figure S1). Higher mortality rates were also seen among non-metropolitan areas and adults 45–64 years old compared to their other respective groups.

### Sensitivity analysis

We found that the increase in mortality shown in the latter years of Fig. 1 is due primarily to mortality in the year 2020, which appears to have increased the APC of the final time-intervals for each subgroup. Figure 1 shows an increase in AAMR above baseline rising dramatically in 2020. Our sensitivity analysis was done on sex by repeating the joinpoint regression excluding 2020 (Fig. 3). It showed a decrease in APCs for each time-interval compared to the main analysis in Fig. 1. The increased AAMR APC in 2020 coincides with the peak onset of the Covid-19 pandemic and is most likely due to indirect effects of pandemic-related changes. This implies increased alcohol-consumption during Covid-19 lock-downs played one of the largest roles in an increase in recent ALD mortality which is also supported by recent studies [1]. Although causality cannot be proven due to our study’s cross-sectional design, the following mortality trends should be interpreted in light of the presumption of Covid-19’s significant effect on mortality.

### Sex-related trends

Other studies have shown that as with most ethnicities AI/AN men have a higher ALD mortality rate than women [2, 12–14], but CDC data in our study further demonstrated that the percent change in mortality has recently risen higher among AI/AN women (at 17.7% and 25.3% for 2003–2008 and 2018–2020 respectively) compared to men. US county-level data on drinking patterns showed that although the prevalence of all levels of alcohol use were nearly equal for males and females (with a median ratio of 1.4 for 2002–2012 data), females were more likely to binge-drink [15], the cause of which is unclear.

### Age-related trends

Although one can visibly see a significant increase in the mortality rate from 2019 onwards for age groups 25–44 and 45–64 years of age, the joinpoint regression model revealed that AI/ANs aged 25–44 saw the greatest increase in mortality rate in 2018–2020 with an APC of 31.0 (95% CI: 7.7–27.7). This trend was likely driven by worsening pandemic changes in the year 2020 for young adults. These findings were consistent with at least one other mortality study on AI/ANs, where although heart disease is the leading cause of mortality for AI/ANs and all racial/ethnic groups overall [16, 17], AI/ANs had unintentional injury and CLD including cirrhosis as the next additional leading causes of death after CVD mortality [14].

Lung cancer is the leading cancer-related mortality cause among AI/ANs, with the largest amount of smoking-attributed mortality found among AI/ANs compared to other racial/ethnic groups. Among lung cancer cases in AI/ANs, 90% were attributed to smoking [17]. This highlights health disparities related to substance use among AI/ANs as a driver of mortality. In a study on reservation-based adolescent American Indian substance abuse, the alcohol use prevalence of eighth graders was 52.8%, second only to marijuana use at 56.2%. Their prevalence rates for all substances were also significantly higher than for other ethnicities [18]. Early onset of drinking is associated with a greater likelihood for alcohol dependence [19, 20], and American Indians are known to begin drinking at a younger age compared to other ethnicities. Their average age at first sip is 12.4 and usually given to them by an adult >21 years old [19–21]. It seems reasonable to infer that the higher APC among the younger age group could be explained by increased binge-drinking social behaviors and the peer pressure towards heavy social drinking. This trend of alcohol use continues among college-aged and working-aged adults. This effect could be worsened during 2020 because the younger age groups are affected most by the social pressures of pandemic changes such as loss of vulnerable jobs. Supporting data is sparse. Further studies are needed targeting younger AI/AN age groups on college campus interventions or qualitative studies on what risk factors make young AI/AN adults more likely to drink.

### Trends by geography

The most populous states for AI/ANs where over 50.9% reside are Oklahoma, Arizona (12.9%), California, New Mexico (9.1%), and Texas based on 2020 census data [22, 23] (Fig. 2). New Mexico and Arizona fell in the 90th percentile of states having >10 times the AAMR of other states. It would be reasonable for policymakers to focus on improving local laws and funding alcohol abuse programs in these key states.

Regional differences could partly be explained by tribal inheritance patterns of alcohol dehydrogenase (ADH) and aldehyde dehydrogenase (ALDH) which metabolize alcohol into acetaldehyde, then into the less toxic acetate, respectively [24, 25]. Studies among Southwest California Indians noted a 6% prevalence of the ADH1B*3 allele, which was associated among Mission Indians with one-third less likelihood to drink alcohol. This could be because this allele leads to an ADH that metabolizes alcohol more rapidly compared to other alleles in the polymorphisms behind these enzymes, leading to facial flushing and symptoms of alcohol intoxication at lower levels of alcohol consumption [12, 24]. Further genome wide association studies specifically on AI/ANs could further elucidate tribes and reservations with other genetic protective polymorphisms.

Paradoxically, there is both a high prevalence of alcohol abstinence and also alcohol use disorder among AI/ANs [26]. Some studies found that while binge drinking among AI/ANs is more common, AI/ANs do not drink more alcohol per day or for longer durations, yet they still suffer more deleterious effects compared to other ethnic groups [27]. Other studies are also needed to correlate specific geographic regions to alleles that make binge-drinking more susceptible in order to inform local laws and enact protective policies such as alcohol taxes, behavioral health services targeting alcoholism, and substance use services in these regions.

### Trends related to level of urbanization

In terms of urbanization, non-metropolitan areas saw a dramatic increase in APC to 25.5 in 2018–2020 (95% CI: 5.0–50.0) not seen in large or medium/small metropolitan areas. As can be seen in Fig. 1, the AAMR per 100,000 deaths increased in every level of urbanization from 2019 to 2020. If the rise in mortality is considered attributable directly to SARS-CoV-2, one would expect a higher percent change in mortality in large metropolitan areas instead of rural areas due to greater viral spread in more densely populated areas. Therefore, the mortality increase is more likely due to the indirect effects of multiple pandemic-related factors.

During the Covid-19 global pandemic, in 2019–2020 AI/ANs saw the highest increase in AAMR at 34.5% compared to all other races and ethnicities [2], with ALD being one of many leading causes for the greatest rise in APC [2, 28]. In 2020, the AAMR in AI/ANs due to ALD rose by 40% compared to pre-pandemic levels. Death certificate data in 2020 showed that although the rate of alcohol-related deaths increased by 25% from 2019–2020 (which was higher than all-cause mortality at 16.6%), only a small portion of alcohol-related deaths was attributed to Covid-19 [5]. Data are limited, but public health surveillance data suggest a high prevalence of hepatitis C on Indian reservations [29, 30], which could be as high as 8.6% [29]. However, this mid-pandemic mortality rise was not seen among CLD mortalities related to other etiologies such as viral hepatitis, demonstrating that alcohol played a specific role in the AAMR apart from other causes, the effect of which was worse among AI/ANs [28].

During the Covid-19 pandemic, US retail alcohol sales increased for all demographic and geographic categories by at least 34% [31], but the increase in mean AAMR during this period was estimated to have risen disproportionately 6 times higher among AI/ANs compared to White individuals [2]. Despite pandemic-related restaurant and bar closures, retail alcohol sales for home consumption increased by 34% for all demographics and geographic categories during the pandemic, and higher stress and anxiety was also reported [31]. Mandated lockdowns and more time spent at home, coupled with increased social stress due to loss of work, could have also increased the propensity for alcohol use. These unique challenges may have affected vulnerable rural areas more than more resilient metropolitan areas.

ALD is usually diagnosed at late stages when there is less time for medical intervention. Perhaps the increased trend in ALD mortality was also due to decreased CLD clinic follow-up, fewer endoscopies, and a lack of lab visits leading to a lower clinical detection of worsening disease. This was further compounded by isolation and quarantine requirements for those who had incident Covid-19, creating an opportunity for increased alcohol use and the sale of alcohol [28].

Urbanization could also influence the prevalence and rate of detection of additional etiologies of concomitant chronic liver disease, as larger metropolitan areas have more clinicians and tertiary hospitals for prevention and recommended screening.

### AI/AN mortality compared to other racial/ethnic groups

Our analysis focused on mortality in AI/ANs and did not compare AI/AN mortality to those of other racial/ethnic groups. However, other studies on CDC WONDER data made this comparison and showed that AI/ANs had a mean AAMR due to ALD nearly six times higher than in the White majority at 68.5 compared to 11.7 per 100,000 [2]. AI/ANs accounted for 9.6% of all ALD deaths although they represented only 3.2% of the total population in 2020 [2]. AI/ANs have the highest death rate from ALD and the highest rates of alcohol-related deaths of all US racial/ethnic groups including NH White, NH Black, and Asian groups [12]. US states with the highest AAMR due to ALD were closely correlated with states that had the highest ALD AAMRs among AI/ANs during the Covid-19 pandemic.

CDC cause-specific life table data from US National Vital Statistics showed that AI/ANs experienced the greatest decline in life expectancy from 2020 to 2021 of all racial and ethnic groups, due primarily to unintentional injuries (24.3%), Covid-19 (21.1%), and CLD including cirrhosis (20.1%) during the pandemic period, whereas mortality due to Covid-19 for all races/ethnicities overall accounted for 59.7% of mortality [32]. Comparatively, the decline in non-Hispanic (NH) White life expectancy was due primarily to Covid-19 (64.0%), heart disease (3.3%), and third CLD including cirrhosis (2.6%).

Based on CDC WONDER data, other studies showed ALD as an underlying cause of death increased from 24,106 to 29,504 (a 22.4% increase) [5]. Another study showed all-cause premature mortality was projected to decline from 1990 to 2016 and continue to decline through 2030 for all common causes of death including chronic liver disease, heart disease, cancer, and suicide, for all US racial groups except non-Hispanic White females and AI/ANs who would see a 10% increase according to Best et al [33]. However, due to the Covid-19 pandemic, as suggested by our study, the actual increase in mortality among AI/ANs is likely much higher than the expected 10%.

Regarding cardiovascular disease, AI/ANs had the highest prevalence of disease for all conditions that are risk factors for heart disease, including a higher prevalence of hyperlipidemia, hypertension, smoking, and diabetes compared to Asians, Hispanics, and African Americans [34].

### Limitations

A strength of our study is in the large sample size of the AI/AN population in CDC WONDER data since many national databases, including data from the nationwide *Healthy People* initiative, do not have adequate numbers to meet statistical reliability or for confidentiality on AI/ANs [34]. Since the consumption of alcohol is associated with over 200 diseases, and may coexist with other causes of liver disease [12], this cross-sectional study is unable to directly link ALD as an underlying cause of mortality in the data. Death certificate data is also a notoriously challenging source from which to reliably derive underlying mortality causes, especially in AI/ANs commonly due to racial/ethnic misclassification due to the small population size [34]. ALD mortality is probably higher than reported in studies since many liver-related mortalities are coded with an unknown etiology on death certificates [35]. The results included only non-Hispanic populations, and further studies would be needed to examine trends comparing Hispanic to non-Hispanic AI/ANs.

### Suggested interventions

Based on these findings of a recent increase in ALD mortality, investigators and policymakers should follow evidence-based approaches proven to be effective at reducing health disparities among AI/ANs. Firstly, cohort studies are needed to further elucidate the risk factors leading to higher pandemic AAMR, and follow-up cross-sectional studies should be done now that the pandemic has abated to determine if mortality has returned to pre-pandemic expected levels when data for post-pandemic years are available. If mortality is still elevated beyond 2021, population-level interventions on pricing policies have been proven to be the most effective short-term interventions to decrease alcohol-related mortality. A 10% price hike led to a 4.4% decrease in alcohol consumption per capita and in 50 studies a 3.5% decline in alcohol-attributable mortality [35].

ALD if detected early is preventable, yet 75% of cases end in premature death due to other sequelae such as cirrhosis or liver cancer [35]. Research efforts should focus on new asymptomatic screens with high sensitivity for early detection, and if effective screens are established, they should focus on the young adult and middle-aged based on our findings. It has been shown that an increase in the minimum legal drinking age leads to less drinking and motor vehicle accidents among young adults [35]. Preventive public health should focus on encouraging abstinence or a reduction in alcohol consumption. Warning labels on alcoholic beverages, school-based programs, and large-scale advertising against alcohol have been shown to be less effective. For public health interventions to be effective, a culturally contextualized approach focusing on prevention is the most reasonable way to tackle health disparities among AI/ANs [36].

## Conclusion

There was an overall steep uptick in ALD mortality rate among women, rural areas, and younger age groups coinciding with the Covid-19 pandemic timeline that commands further studies. Our study focuses on alarming trends in ALD mortality rates, highlighting lesser-known risk factors of a known health disparity among US AI/ANs as a vulnerable minority group. These alarming trends are a call to action to investigate the underlying causality for the recent increases in AAMR with further studies to determine a place for targeted interventions.

## Supplementary Information

Supplementary Material 1.

## Data Availability

The datasets generated and analyzed during the current study are publicly accessible from the website for the Centers for Disease Control and Prevention National Center for Health Statistics. The datasets supporting the conclusions of this article are available in the CDC WONDER database publicly available on the CDC WONDER website – https://wonder.cdc.gov/

## Abbreviations

AAMR: Age-adjusted mortality rate
AAPC: Average annual percent change
ADH: Alcohol dehydrogenase
AI/ANs: Non-Hispanic American Indian or Alaskan Natives
ALD: Alcohol-associated liver disease
ALDH: Aldehyde dehydrogenase
APC: Annual percent change
CDC WONDER: Centers for Disease Control and Prevention Wide-Ranging Online Data for Epidemiologic Research
CI: Confidence Interval
CLD: Chronic liver disease
CMRs: Crude mortality rates
CVD: Cardiovascular disease
US, USA: United States of America
