# Supplementary material for: Trends in alcohol-associated liver disease mortality rates in American Indians and Alaskan Natives
Source: BMC Public Health. 2025 Jun 2;25:2046. doi: 10.1186/s12889-025-22895-x (PMC12128309; doi:10.1186/s12889-025-22895-x)
Supplement: Supplementary file 1 — Supplementary Material 1. [file 12889_2025_22895_MOESM1_ESM.docx]

**Supplemental Figures**


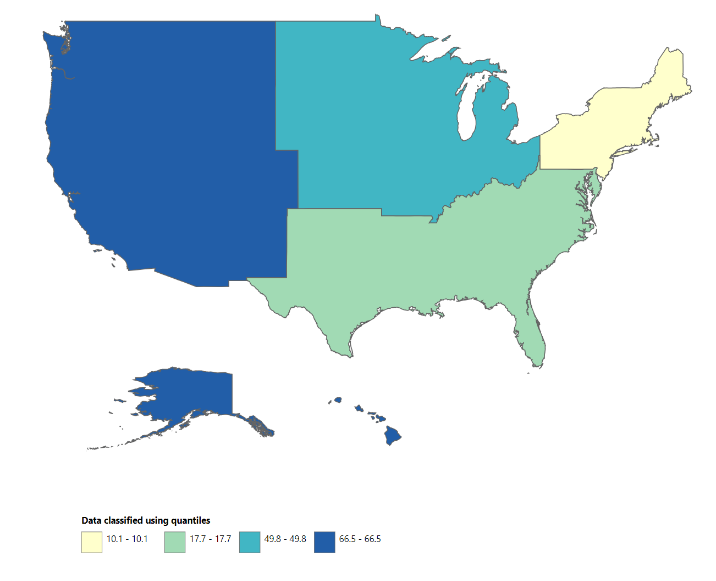


**Supplemental Figure S1** – Map of the United States for alcohol-associated liver disease (ALD), stratified by state in American Indian and Alaskan Natives (AI/ANs), by quantiles of age-adjusted mortality rate (AAMR), 1999 to 2020, stratified by census region according to the 2013 US census classification.

**Supplemental Figure S2** – This figure shows mortality trends among American Indian and Alaskan Natives (AI/ANs) by cause of death, comparing all-cause and cardiovascular disease (CVD) mortality to that caused by alcohol-associated liver disease. CVD mortality has a higher mortality rate than ALD mortality, but the annual percentage change (APC) in 2018-2020 is higher at 16.4% vs 10.7%.

**Supplemental Tables**

**Supplementary Table S1:** Age-adjusted Mortality Rates of Alcohol-Associated Liver Disease Mortality Stratified by Sex in American Indian/Alaskan Natives, 1999-2020

|  | **Females** | | | **Males** | | |
| --- | --- | --- | --- | --- | --- | --- |
| **Year** | **Age-Adjusted Mortality Rate/100,000** | **Age-Adjusted**  **95% CI** | **Age-Adjusted Standard Error** | **Age-Adjusted Mortality Rate/100,000** | **Age-Adjusted**  **95% CI** | **Age-Adjusted Standard Error** |
| 1999 | 20.0 | (16.5-23.5) | 1.8 | 36.1 | (30.5, 41.7) | 2.8 |
| 2000 | 15.5 | (12.4-18.6) | 1.6 | 22.6 | (18.4, 26.8) | 2.1 |
| 2001 | 16.1 | (13.2-19.1) | 1.5 | 26.2 | (21.8, 30.6) | 2.2 |
| 2002 | 14.9 | (12.0-17.8) | 1.5 | 26.7 | (22.5, 30.9) | 2.1 |
| 2003 | 16.0 | (13.0-19.0) | 1.5 | 25.5 | (21.6, 29.4) | 2.0 |
| 2004 | 17.7 | (14.7-20.8) | 1.6 | 32.8 | (28.1, 37.6) | 2.4 |
| 2005 | 17.8 | (14.7-20.9) | 1.6 | 36.2 | (31.4, 40.9) | 2.4 |
| 2006 | 24.9 | (21.2-28.5) | 1.8 | 39.3 | (34.5, 44.1) | 2.4 |
| 2007 | 29.3 | (25.4-33.1) | 2.0 | 51.1 | (45.6, 56.5) | 2.8 |
| 2008 | 33.9 | (29.7-38.1) | 2.1 | 49.8 | (44.3, 55.2) | 2.8 |
| 2009 | 31.8 | (27.8-35.8) | 2.0 | 45.7 | (40.8, 50.7) | 2.5 |
| 2010 | 34.5 | (30.4-38.6) | 2.1 | 57.5 | (51.8, 63.1) | 2.9 |
| 2011 | 36.2 | (32.0-40.4) | 2.1 | 54.4 | (48.9, 59.9) | 2.8 |
| 2012 | 39.9 | (35.5-44.2) | 2.2 | 60.8 | (55.1, 66.6) | 2.9 |
| 2013 | 38.7 | (34.4-43.0) | 2.2 | 63.8 | (58.1, 69.5) | 2.9 |
| 2014 | 44.3 | (39.8-48.9) | 2.3 | 59.6 | (54.1, 65.1) | 2.8 |
| 2015 | 45.3 | (40.7-49.9) | 2.4 | 69.3 | (63.4, 75.1) | 3.0 |
| 2016 | 44.9 | (40.4-49.4) | 2.3 | 68.0 | (62.2, 73.7) | 2.9 |
| 2017 | 45.5 | (40.9-50.0) | 2.3 | 69.4 | (63.6, 75.2) | 2.9 |
| 2018 | 48.9 | (44.2-53.6) | 2.4 | 74.9 | (68.9, 80.9) | 3.1 |
| 2019 | 53.8 | (48.8-58.8) | 2.5 | 68.7 | (63.0, 74.5) | 2.9 |
| 2020 | 78.5 | (72.5-84.5) | 3.1 | 99.3 | (92.4, 106.2) | 3.5 |

CI = Confidence Interval.

**Supplementary Table S2:** Age-adjusted and Crude Mortality Rates of Alcohol-Associated Liver Disease Mortality Stratified by Age Groups in American Indian/Alaskan Natives, 1999-2020

| **Age Group, y** | **Year** | **Total Deaths (N) per 100,000** | **Crude Mortality Rate (CMR) per 100,000** | **Age-Adjusted Mortality Rate (AAMR) per 100,000** | **Age-Adjusted**  **95% CI** | **Age-Adjusted Standard Error** | **% of Total Deaths** |
| --- | --- | --- | --- | --- | --- | --- | --- |
| 25-44 |  |  |  |  |  |  |  |
|  | 1999 | 129 | 18.6 | 19.2 | (15.9, 22.5) | 1.7 | 2.80% |
|  | 2000 | 91 | 12.8 | 13.2 | (10.6, 16.1) | 1.4 | 1.90% |
|  | 2001 | 98 | 14.0 | 14.3 | (11.6, 17.4) | 1.4 | 2.10% |
|  | 2002 | 102 | 14.7 | 15.1 | (12.1, 18.0) | 1.5 | 2.20% |
|  | 2003 | 98 | 14.3 | 14.7 | (11.9, 17.9) | 1.5 | 2.10% |
|  | 2004 | 104 | 15.2 | 15.7 | (12.7, 18.7) | 1.5 | 2.20% |
|  | 2005 | 117 | 17.2 | 17.7 | (14.5, 20.9) | 1.6 | 2.50% |
|  | 2006 | 132 | 19.5 | 20.1 | (16.7, 23.5) | 1.8 | 2.80% |
|  | 2007 | 175 | 25.8 | 26.9 | (22.9, 30.9) | 2.0 | 3.70% |
|  | 2008 | 191 | 28.1 | 29.3 | (25.2, 33.5) | 2.1 | 4.10% |
|  | 2009 | 177 | 26.0 | 27.3 | (23.3, 31.3) | 2.1 | 3.80% |
|  | 2010 | 221 | 32.3 | 34.7 | (30.1, 39.3) | 2.3 | 4.70% |
|  | 2011 | 214 | 31.2 | 33.2 | (28.7, 37.7) | 2.3 | 4.60% |
|  | 2012 | 227 | 32.8 | 35.1 | (30.5, 39.7) | 2.4 | 4.90% |
|  | 2013 | 243 | 34.8 | 37.6 | (32.8, 42.3) | 2.4 | 5.20% |
|  | 2014 | 259 | 36.7 | 39.6 | (34.7, 44.4) | 2.5 | 5.50% |
|  | 2015 | 273 | 38.1 | 41.3 | (36.4, 46.3) | 2.5 | 5.80% |
|  | 2016 | 284 | 39.0 | 41.8 | (36.9, 46.8) | 2.5 | 6.10% |
|  | 2017 | 290 | 39.2 | 42.8 | (37.8, 47.8) | 2.6 | 6.20% |
|  | 2018 | 321 | 42.7 | 45.4 | (40.4, 50.5) | 2.6 | 6.90% |
|  | 2019 | 353 | 46.2 | 49.8 | (44.5, 55.0) | 2.7 | 7.50% |
|  | 2020 | 581 | 75.1 | 80.4 | (73.8, 87.1) | 3.4 | 12.40% |
| 45-64 |  |  |  |  |  |  |  |
|  | 1999 | 156 | 37.2 | 37.5 | (31.6, 43.4) | 3.0 | 1.80% |
|  | 2000 | 113 | 25.5 | 25.6 | (20.9, 30.4) | 2.4 | 1.30% |
|  | 2001 | 143 | 30.8 | 30.7 | (25.7, 35.7) | 2.6 | 1.70% |
|  | 2002 | 135 | 27.9 | 27.8 | (23.1, 32.5) | 2.4 | 1.60% |
|  | 2003 | 163 | 32.3 | 32.3 | (27.3, 37.2) | 2.5 | 1.90% |
|  | 2004 | 197 | 37.6 | 37.7 | (32.5, 43.0) | 2.7 | 2.30% |
|  | 2005 | 223 | 41.0 | 41.0 | (35.6, 46.4) | 2.7 | 2.60% |
|  | 2006 | 268 | 47.7 | 47.6 | (41.9, 53.3) | 2.9 | 3.20% |
|  | 2007 | 351 | 60.6 | 60.6 | (54.3, 67.0) | 3.2 | 4.20% |
|  | 2008 | 357 | 60.0 | 59.9 | (53.7, 66.1) | 3.2 | 4.20% |
|  | 2009 | 367 | 60.1 | 60.2 | (54.1, 66.4) | 3.1 | 4.30% |
|  | 2010 | 420 | 67.7 | 67.8 | (61.3, 74.3) | 3.3 | 5.00% |
|  | 2011 | 427 | 67.2 | 67.6 | (61.1, 74.0) | 3.3 | 5.10% |
|  | 2012 | 472 | 73.8 | 74.6 | (67.9, 81.4) | 3.4 | 5.60% |
|  | 2013 | 497 | 77.3 | 77.7 | (70.9, 84.6) | 3.5 | 5.90% |
|  | 2014 | 500 | 77.2 | 77.1 | (70.3, 83.9) | 3.5 | 5.90% |
|  | 2015 | 573 | 88.0 | 88.4 | (81.1, 95.7) | 3.7 | 6.80% |
|  | 2016 | 548 | 83.7 | 84.0 | (76.9, 91.2) | 3.6 | 6.50% |
|  | 2017 | 570 | 87.0 | 88.0 | (80.6, 95.3) | 3.8 | 6.80% |
|  | 2018 | 602 | 92.1 | 92.1 | (84.6, 99.7) | 3.8 | 7.10% |
|  | 2019 | 573 | 88.1 | 89.5 | (82.0, 97.0) | 3.8 | 6.80% |
|  | 2020 | 788 | 121.8 | 123 | (114.2, 131.9) | 4.5 | 9.30% |
| ≥65 |  |  |  |  |  |  |  |
|  | 1999 | 38 | 29.2 | 27.9 | (19.7, 38.5) | 4.6 | 2.20% |
|  | 2000 | 28 | 20.0 | 19.7 | (13.0, 28.6) | 3.8 | 1.60% |
|  | 2001 | 29 | 20.2 | 18.2 | (12.1, 26.3) | 3.4 | 1.70% |
|  | 2002 | 34 | 23.0 | 20.3 | (14.0, 28.6) | 3.5 | 2.00% |
|  | 2003 | 24 | 15.7 | 14.2 | (9.0, 21.3) | 2.9 | 1.40% |
|  | 2004 | 38 | 24.1 | 23.1 | (16.2, 32.0) | 3.8 | 2.20% |
|  | 2005 | 37 | 22.5 | 21.0 | (14.7, 29.1) | 3.5 | 2.20% |
|  | 2006 | 60 | 35.2 | 31.1 | (23.6, 40.2) | 4.1 | 3.50% |
|  | 2007 | 65 | 36.7 | 33.1 | (25.4, 42.3) | 4.2 | 3.80% |
|  | 2008 | 70 | 37.9 | 36.5 | (28.2, 46.5) | 4.5 | 4.10% |
|  | 2009 | 56 | 29.1 | 26.4 | (19.8, 34.6) | 3.6 | 3.30% |
|  | 2010 | 67 | 33.7 | 30.7 | (23.6, 39.2) | 3.8 | 3.90% |
|  | 2011 | 70 | 33.1 | 31.1 | (24.1, 39.6) | 3.8 | 4.10% |
|  | 2012 | 95 | 42.1 | 40.3 | (32.4, 49.6) | 4.3 | 5.60% |
|  | 2013 | 91 | 37.9 | 34.0 | (27.2, 41.9) | 3.6 | 5.30% |
|  | 2014 | 96 | 37.7 | 34.2 | (27.6, 42.0) | 3.6 | 5.60% |
|  | 2015 | 105 | 38.9 | 37.0 | (29.7, 44.3) | 3.7 | 6.20% |
|  | 2016 | 127 | 44.6 | 40.0 | (32.8, 47.1) | 3.6 | 7.40% |
|  | 2017 | 120 | 40.0 | 35.2 | (28.8, 41.7) | 3.3 | 7.00% |
|  | 2018 | 149 | 47.1 | 43.9 | (36.7, 51.1) | 3.7 | 8.70% |
|  | 2019 | 131 | 39.4 | 36.4 | (30.0, 42.8) | 3.3 | 7.70% |
|  | 2020 | 175 | 49.9 | 46.5 | (39.5, 53.6) | 3.6 | 10.30% |

CI = Confidence Interval. y = years of age.

**Supplementary Table S3:** Age-adjusted and Crude Mortality Rates of Alcohol-Associated Liver Disease Mortality Stratified in American Indian/Alaskan Natives by Level of Urbanization, 1999-2020

| **Level of Urbanization** | **Year** | **Total Deaths (N) per 100,000** | **Crude Mortality Rate (CMR) per 100,000** | **Age-Adjusted Mortality Rate (AAMR) per 100,000** | **Age-Adjusted**  **95% CI** | **Age-Adjusted Standard Error** | **% of Total Deaths** |
| --- | --- | --- | --- | --- | --- | --- | --- |
| Large Metropolitan |  |  |  |  |  |  |  |
|  | 1999 | 82 | 21.1 | 22.3 | (17.5, 28.0) | 2.6 | 2.50% |
|  | 2000 | 58 | 14.5 | 15.8 | (11.8, 20.8) | 2.2 | 1.70% |
|  | 2001 | 60 | 14.8 | 14.6 | (11.1, 18.9) | 1.9 | 1.80% |
|  | 2002 | 65 | 15.9 | 16.2 | (12.4, 20.9) | 2.1 | 2.00% |
|  | 2003 | 73 | 17.7 | 17.9 | (13.8, 22.7) | 2.2 | 2.20% |
|  | 2004 | 86 | 20.6 | 19.8 | (15.7, 24.5) | 2.2 | 2.60% |
|  | 2005 | 84 | 19.7 | 19.9 | (15.7, 24.8) | 2.2 | 2.50% |
|  | 2006 | 113 | 26.2 | 26.1 | (21.2, 31.1) | 2.5 | 3.40% |
|  | 2007 | 126 | 29.0 | 27.8 | (22.8, 32.8) | 2.6 | 3.80% |
|  | 2008 | 148 | 33.5 | 32.5 | (27.1, 38.0) | 2.8 | 4.50% |
|  | 2009 | 152 | 34.2 | 32.1 | (26.9, 37.4) | 2.7 | 4.60% |
|  | 2010 | 178 | 40.1 | 39.1 | (33.2, 45.0) | 3.0 | 5.40% |
|  | 2011 | 158 | 34.4 | 32.8 | (27.5, 38.1) | 2.7 | 4.80% |
|  | 2012 | 184 | 39.5 | 37.8 | (32.2, 43.5) | 2.9 | 5.50% |
|  | 2013 | 172 | 36.4 | 34.3 | (29.1, 39.5) | 2.7 | 5.20% |
|  | 2014 | 211 | 43.7 | 41.7 | (35.9, 47.5) | 3.0 | 6.40% |
|  | 2015 | 219 | 44.5 | 43.2 | (37.4, 49.1) | 3.0 | 6.60% |
|  | 2016 | 205 | 41.1 | 39.1 | (33.6, 44.6) | 2.8 | 6.20% |
|  | 2017 | 233 | 45.9 | 43.5 | (37.8, 49.2) | 2.9 | 7.00% |
|  | 2018 | 228 | 44.2 | 42.7 | (37.0, 48.4) | 2.9 | 6.90% |
|  | 2019 | 218 | 41.8 | 41.5 | (35.9, 47.1) | 2.9 | 6.60% |
|  | 2020 | 269 | 50.7 | 50.9 | (44.7, 57.2) | 3.2 | 8.10% |
| Medium/Small Metropolitan |  |  |  |  |  |  |  |
|  | 1999 | 81 | 20.9 | 22.5 | (17.6, 28.3) | 2.6 | 1.80% |
|  | 2000 | 71 | 17.7 | 18.3 | (14.1, 23.4) | 2.3 | 1.60% |
|  | 2001 | 80 | 19.7 | 19.9 | (15.6, 24.9) | 2.3 | 1.80% |
|  | 2002 | 76 | 18.3 | 19.1 | (14.9, 24.1) | 2.3 | 1.70% |
|  | 2003 | 79 | 18.7 | 18.0 | (14.2, 22.5) | 2.1 | 1.70% |
|  | 2004 | 96 | 22.3 | 21.9 | (17.6, 26.8) | 2.3 | 2.10% |
|  | 2005 | 111 | 25.2 | 24.9 | (20.1, 29.8) | 2.5 | 2.50% |
|  | 2006 | 130 | 28.9 | 28.2 | (23.2, 33.2) | 2.5 | 2.90% |
|  | 2007 | 186 | 40.5 | 39.7 | (33.9, 45.6) | 3.0 | 4.10% |
|  | 2008 | 169 | 36.0 | 34.6 | (29.3, 40.0) | 2.7 | 3.70% |
|  | 2009 | 176 | 36.8 | 34.9 | (29.6, 40.1) | 2.7 | 3.90% |
|  | 2010 | 203 | 41.8 | 40.5 | (34.8, 46.2) | 2.9 | 4.50% |
|  | 2011 | 219 | 44.4 | 42.3 | (36.6, 48.0) | 2.9 | 4.80% |
|  | 2012 | 237 | 47.2 | 46.6 | (40.5, 52.7) | 3.1 | 5.20% |
|  | 2013 | 275 | 54.0 | 52.6 | (46.2, 58.9) | 3.2 | 6.10% |
|  | 2014 | 263 | 50.7 | 50.2 | (44.0, 56.4) | 3.2 | 5.80% |
|  | 2015 | 285 | 54.1 | 52.4 | (46.2, 58.7) | 3.2 | 6.30% |
|  | 2016 | 301 | 56.1 | 54.5 | (48.2, 60.8) | 3.2 | 6.70% |
|  | 2017 | 308 | 56.0 | 56.0 | (49.6, 62.4) | 3.3 | 6.80% |
|  | 2018 | 354 | 63.3 | 61.6 | (55.0, 68.1) | 3.4 | 7.80% |
|  | 2019 | 326 | 57.3 | 57.5 | (51.1, 63.9) | 3.3 | 7.20% |
|  | 2020 | 496 | 85.7 | 86.2 | (78.4, 94.0) | 4.0 | 11.00% |
| Non-Metropolitan |  |  |  |  |  |  |  |
|  | 1999 | 160 | 34.2 | 35.1 | (29.5, 40.7) | 2.9 | 2.30% |
|  | 2000 | 103 | 20.9 | 21.4 | (17.2, 25.7) | 2.2 | 1.50% |
|  | 2001 | 130 | 26.2 | 26.2 | (21.7, 30.8) | 2.3 | 1.90% |
|  | 2002 | 130 | 25.9 | 25.2 | (20.8, 29.6) | 2.2 | 1.90% |
|  | 2003 | 133 | 26.2 | 25.1 | (20.8, 29.4) | 2.2 | 1.90% |
|  | 2004 | 157 | 30.4 | 30.6 | (25.7, 35.5) | 2.5 | 2.20% |
|  | 2005 | 182 | 34.9 | 33.3 | (28.4, 38.2) | 2.5 | 2.60% |
|  | 2006 | 217 | 40.9 | 39.3 | (34.0, 44.7) | 2.7 | 3.10% |
|  | 2007 | 279 | 51.6 | 49.3 | (43.5, 55.2) | 3.0 | 4.00% |
|  | 2008 | 301 | 54.8 | 53.7 | (47.5, 59.9) | 3.2 | 4.30% |
|  | 2009 | 272 | 48.3 | 46.4 | (40.8, 52.0) | 2.9 | 3.90% |
|  | 2010 | 327 | 57.0 | 54.5 | (48.5, 60.5) | 3.1 | 4.70% |
|  | 2011 | 334 | 57.6 | 56.0 | (49.9, 62.2) | 3.1 | 4.80% |
|  | 2012 | 373 | 63.3 | 61.9 | (55.5, 68.3) | 3.3 | 5.30% |
|  | 2013 | 384 | 64.1 | 62.2 | (55.8, 68.5) | 3.2 | 5.50% |
|  | 2014 | 381 | 62.7 | 60.1 | (54.0, 66.3) | 3.1 | 5.50% |
|  | 2015 | 447 | 72.3 | 71.3 | (64.5, 78.1) | 3.5 | 6.40% |
|  | 2016 | 453 | 71.7 | 70.7 | (64.1, 77.4) | 3.4 | 6.50% |
|  | 2017 | 439 | 68.8 | 68.4 | (61.8, 75.0) | 3.3 | 6.30% |
|  | 2018 | 490 | 75.7 | 75.6 | (68.7, 82.5) | 3.5 | 7.00% |
|  | 2019 | 513 | 78.2 | 79.4 | (72.3, 86.5) | 3.6 | 7.30% |
|  | 2020 | 779 | 117.7 | 121.2 | (112.4, 129.9) | 4.5 | 11.20% |
